# Landscape genetics identifies streams and drainage infrastructure as dispersal corridors for an endangered wetland bird

**DOI:** 10.1002/ece3.4296

**Published:** 2018-07-24

**Authors:** Charles B. van Rees, J. Michael Reed, Robert E. Wilson, Jared G. Underwood, Sarah A. Sonsthagen

**Affiliations:** ^1^ Department of Biology Tufts University Medford Massachusetts; ^2^ U. S. Geological Survey Alaska Science Center Anchorage Alaska; ^3^ Pacific Reefs National Wildlife Refuge Complex U.S. Fish and Wildlife Service Honolulu Hawaii; ^4^ Present address: Don Edwards San Francisco Bay National Wildlife Refuge Fremont California

**Keywords:** connectivity, Hawaii, landscape resistance, metapopulation, Moorhen, Waterbird

## Abstract

Anthropogenic alterations to landscape structure and composition can have significant impacts on biodiversity, potentially leading to species extinctions. Population‐level impacts of landscape change are mediated by animal behaviors, in particular dispersal behavior. Little is known about the dispersal habits of rails (Rallidae) due to their cryptic behavior and tendency to occupy densely vegetated habitats. The effects of landscape structure on the movement behavior of waterbirds in general are poorly studied due to their reputation for having high dispersal abilities. We used a landscape genetic approach to test hypotheses of landscape effects on dispersal behavior of the Hawaiian gallinule (*Gallinula galeata sandvicensis*), an endangered subspecies endemic to the Hawaiian Islands. We created a suite of alternative resistance surfaces representing biologically plausible a priori hypotheses of how gallinules might navigate the landscape matrix and ranked these surfaces by their ability to explain observed patterns in genetic distance among 12 populations on the island of O`ahu. We modeled effective distance among wetland locations on all surfaces using both cumulative least‐cost‐path and resistance‐distance approaches and evaluated relative model performance using Mantel tests, a causal modeling approach, and the mixed‐model maximum‐likelihood population‐effects framework. Across all genetic markers, simulation methods, and model comparison metrics, surfaces that treated linear water features like streams, ditches, and canals as corridors for gallinule movement outperformed all other models. This is the first landscape genetic study on the movement behavior of any waterbird species to our knowledge. Our results indicate that lotic water features, including drainage infrastructure previously thought to be of minimal habitat value, contribute to habitat connectivity in this listed subspecies.

## 1 INTRODUCTION

Research on animal movement behavior, in particular how landscape features affect dispersal, is essential for predicting, understanding, and managing the impacts of ongoing changes in climate and landscape structure on animal populations (Hanski, 2001; Holyoak & Heath, 2016; Knowlton & Graham, 2010; van Strien et al., 2014). Although direct data on animal movement can be time‐consuming and expensive to collect, the development of indirect methods using genetic markers to estimate rates of dispersal has greatly increased understanding of population connectivity (Anderson, Kierepka, Swihart, Latch, & Rhodes, 2015; Epps, Wehausen, Bleich, Torres, & Brashares, 2007; Lowe & Allendorf, 2010). These indices can be especially important for studying the movement of behaviorally cryptic species that are difficult to study through other means like mark–resighting (Finnegan et al., 2012), and provide estimates of genetic differentiation due to dispersal and subsequent gene flow (Sexton, Hangartner, & Hoffmann, 2014; Waser & Strobeck, 1998).

The field of landscape genetics provides an analytical framework to assess the potential effects of landscape structure and composition on genetic differentiation in wildlife populations (Manel & Holderegger, 2013; Manel, Schwartz, Luikart, & Taberlet, 2003). According to the most basic landscape genetic model, isolation by distance, genetic similarity among populations (or individuals) is correlated with geographic or Euclidean distance (Wright, 1943). The concept of effective distance extends this model by incorporating information on the movement behavior of an organism and by considering distances between points or populations in addition to the permeability of the intervening landscape matrix to movement for that particular organism (Adriaensen et al., 2003; McRae, 2006). Effective distances are quantified using resistance surfaces, spatially explicit models that reflect hypotheses about the degree to which specific landscape features or cover types impede or facilitate movement in a raster format (Spear, Balkenhol, Fortin, McRae, & Scribner, 2010; Storfer et al., 2007). Among a suite of surfaces, those that best explain data on spatial genetic variation are assumed to represent the most likely representation of how a given set of landscape features affects movement in an organism (Cushman & Landguth, 2010; Zeller, McGarigal, & Whiteley, 2012).

Current research using this framework is generally biased toward temperate climates and forest ecosystems on large continents (Balkenhol, Cushman, Waits, & Storfer, 2015; Waits, Cushman, & Spear, 2016). However, avian taxa are underrepresented in these studies (Haig et al., 2011; Kozakiewicz, Carver, & Burridge, 2017; Zeller et al., 2012). Behavioral studies on birds in human‐altered landscapes have demonstrated that the effects of landscape change on movement rates are typically species specific (Fahrig, 2007). Thus, landscape genetic analyses of threatened and endangered bird taxa are urgently needed for a better understanding of the impacts of continued anthropogenic landscape change. Among avian taxa, rails (family Rallidae) are among the most poorly understood with regard to their movement ecology, due to their cryptic behavior and tendency to inhabit densely vegetated habitats (Ripley, Lansdowne, & Olson, 1977; Taylor, 2010). Rails also exhibit the interesting behavioral–evolutionary tendency to colonize widespread and isolated islands or habitat patches, while appearing to have a natural antipathy to disperse after colonization, often becoming flightless (Livezey, 2003; Steadman, 2006). Coupling these behaviors with the discrete and naturally fragmented nature of many wetland ecosystems, and further isolation by anthropogenic landscape change, wetland‐specialist birds like rails are a convenient study system for landscape genetic research. The sensitivity of wetland ecosystems to a diversity of anthropogenic threats (Green et al., 2017; Strayer & Dudgeon, 2010) makes wetland birds a group for which landscape genetic research is likely of substantial importance to conservation.

**Figure 1:**
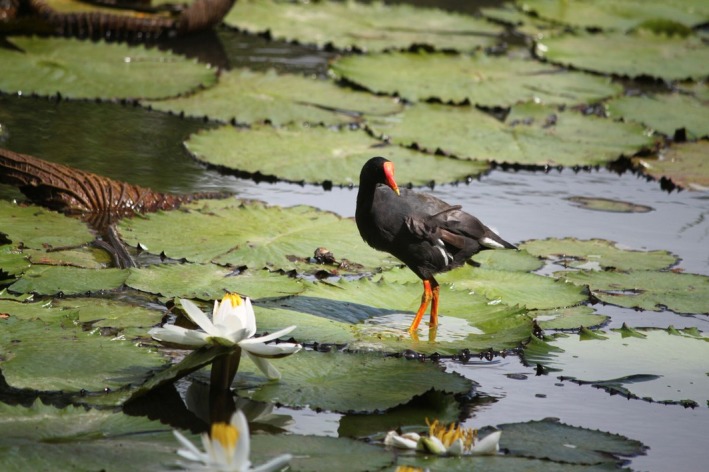
An adult Hawaiian gallinule stands on lilly pads at in a golf course water hazard in Kailua, Hawai`i. Photograph credit Amanda Sandor

Our interest is in one member of the Rallidae, the Hawaiian gallinule (*Gallinula galeata sandvicensis*, Figure 1), which is an endangered subspecies of the common gallinule endemic to freshwater wetlands of the Hawaiian Islands (United States; Bannor & Kiviat, 2002). Hawaiian gallinules were once found on the five main Hawaiian Islands, but were extirpated from all islands other than O`ahu and Kauai during the late 19th to mid‐20th century (Banko, 1987). Habitat loss from anthropogenic landscape change and exotic, invasive wetland plants, as well as predation from introduced mammalian predators drove severe population declines and reductions in the subspecies’ range (Griffin, Shallenberger, Fefer, Sharitz, & Gibbons, 1989; USFWS, 1977). This precipitous decline was eventually halted with legal protection, the establishment of National Wildlife Refuges and state protected areas on these two islands, and the advent of habitat management (predator control and vegetation restoration). The implementation of the latter is associated with a slow but consistent recovery of Hawaii's endangered waterbirds over the last 50 years (Reed, Elphick, Ieno, & Zuur, 2011; Reed, Elphick, Zuur, Ieno, & Smith, 2007; Schwartz & Schwartz, 1949; Underwood, Silbernagle, Nishimoto, & Uyehara, 2013). O`ahu's current gallinule population consists of between 250 and 350 individuals scattered among relict and fragmented wetlands, which are isolated by severe wetland loss on the island (van Rees & Reed, 2014; USFWS, 2011). Wetland habitats on O`ahu are distributed with varying degrees of geographic isolation and are embedded in a complex landscape mosaic of anthropogenic land cover, including highways, recreational areas (e.g., golf courses and resorts), military bases, agricultural land, and residential areas. Connectivity among these fragmented subpopulations is considered an important factor in the conservation and management of Hawaiian gallinules (Reed, DesRochers, VanderWerf, & Scott, 2012a,b; van Rees, Reed, Wilson, Underwood, & Sonsthagen, 2018; Underwood et al., 2013; US Fish and Wildlife Service, 2011). However, little is known about the movement behavior of Hawaiian gallinules, and their cryptic behavior leads to poor detection rates, limiting the efficacy of mark–resight studies (DesRochers, Gee, & Reed, 2008).

Using information from expert opinion and published and unpublished literature, we identified several landscape characteristics and features that may influence movement and gene flow in Hawaiian gallinules. These included roads, urban land cover, (K. Doyle, Hawaii Division of Forestry and Wildlife, pers. comm.), forested areas, steep slopes, and high elevation terrain (Banko, 1987; Perkins, 1903) as potential barriers. By contrast, we expected that mesic areas and open habitats would promote dispersal and gene flow. We specifically predicted that streams and rivers might facilitate dispersal, given reports from a related species and anecdotal observations from experts in this taxon (Nagata, 1983; Takano & Haig, 2004).

This information made it clear that there are a great many alternative explanations for how Hawaiian gallinules might perceive landscape features during dispersal between wetlands. Consequently, we generated a suite of resistance surfaces of the O`ahu landscape matching our proposed hypotheses about the relative resistance of both natural and anthropogenic landscape features to gallinule movement across the landscape. The models are based on observations and biologically informed speculation about distributions and movements of Hawaiian gallinules across the landscape. Once these models were created, we simulated the movement of gallinules across the landscape according to these hypotheses and compared their fits to data on microgeographic genetic differentiation of Hawaiian gallinules on O`ahu (pairwise F_ST_ using microsatellite markers; van Rees, Reed et al., 2018). Our goal was to determine the relationships among landscape features and observed genetic differentiation to identify landscape features that are important to maintaining connectivity among Hawaiian gallinule populations. Results from this study will provide important information for the subspecies’ recovery and to predicting the potential vulnerability of Hawaiian gallinules to future modifications to the landscape as attributable to land use and climate change.

## 2 METHODS

### 2.1 Study area

**Figure 2:**
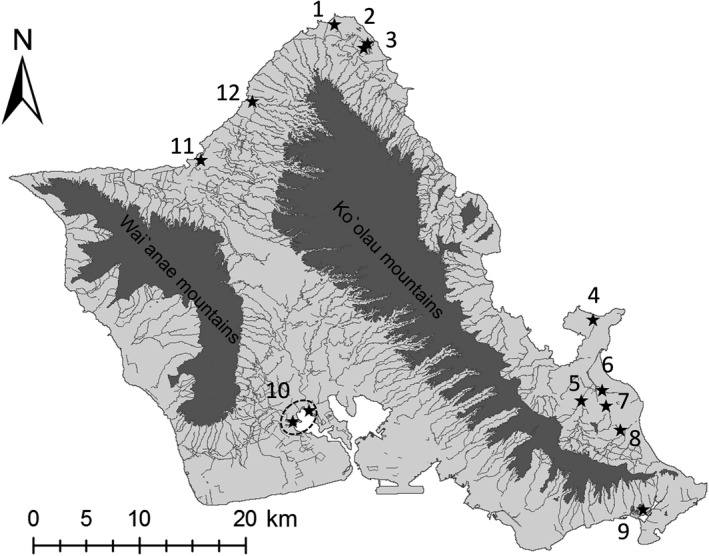
Map of the island of O`ahu, showing locations of the 12 populations sampled for genotyping by van Rees, Reed et al. (2018). Mountain ranges and waterways are pictured in dark gray. Population names are as follows: (1) Turtle Bay resorts, (2) James Campbell National Wildlife Refuge, (3) Kahuku Shrimp Farms, (4) Marine Corps Base Kaneohe, (5) Kawainui Marsh, (6) Hamakua Marsh, (7) Enchanted Lakes, (8) Olomana Golf Links, (9) Keawawa wetland, (10) Pearl harbor (composed of Pouhala Marsh and Pearl Harbor National Wildlife Refuge, Hono`uli`uli unit), (11) Private lotus farm, and (12) Waimea Valley

We studied Hawaiian gallinules on the island of O`ahu (Hawai`i, USA, 21.3156 N, ‐157.858 W), one of two islands that make up the subspecies’ entire range. We collected genetic samples at 12 locations on the island, which represent all known major wetland habitats for the subspecies on O`ahu (Figure 2). Sampled sites were distributed across the island's low‐elevation coastal plain and included sites in Pearl Harbor (site 10 in Figure 2), Honolulu (Keawawa wetlands, site 9), the North shore (Turtle Bay Resort, Kahuku shrimp farms, and James Campbell national Wildlife Refuge, sites 1–3), Hale`iwa town (Waimea valley and a private lotus farm, sites 11 and 12), and the Windward side (Marine Corps Base Kaneohe, Kawainui marsh, Hamakua marsh, Enchanted Lakes, and Olomana Golf Links, sites 4–8).

### 2.2 Genetic data

We obtained multilocus genotypes for 152 Hawaiian gallinules at 12 wetlands from a previous study (van Rees, Reed et al., 2018; mtDNA sequences are accessioned in GenBank, MF673902‐MF673904). We defined wetlands as complexes of spatially proximate and hydrologically linked water bodies. The sampled wetlands included all major breeding areas for the Hawaiian gallinule on the island, and our sample accounts for at a minimum 30% of the known population of the island (Reed et al., 2011; US Fish and Wildlife Service, 2011). We captured gallinules using walk‐in cage traps baited with attractive food items (fresh fruit and cracked corn) and extracted DNA from 4 to 6 body feathers collected from each captured bird. We obtained estimates of interpopulation genetic variance (F_ST_) among the 12 wetland sites from van Rees, Reed et al. (2018); these estimates were based on microsatellite genotype data collected from 12 autosomal loci and 520 base pairs (bp) of the NADH dehydrogenase 2 (ND2) region of mitochondrial DNA (mtDNA). All microsatellite loci were tested for Hardy–Weinberg equilibrium and linkage disequilibrium prior to analysis. Information on genetic diversity, population structure, and analysis of genetic data can be found in van Rees, Reed et al. (2018).

### 2.3 Landscape variables

**Figure 3:**
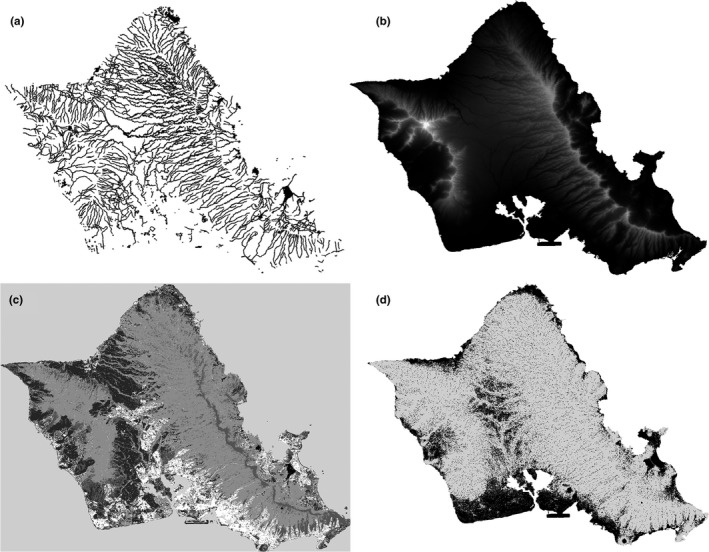
Four example resistance surfaces derived from different spatial datasets. Darker pixels have lower resistance, and lighter pixels have higher resistance. (a) Proximity‐to‐Water, 100‐m corridor. (b) Linear elevation, version A. (c) Land use with all land use classes. (d) TWI two‐class threshold model, version A

We represented landscape variables and movement capacities using resistance surfaces, in which landscapes are modeled as a raster grid, assigning different resistance values to landscape cover classes or features according to the hypothesized difficulty of passing through such features (Spear et al., 2010; Figure 3). We analyzed 20 resistance surfaces that addressed 10 hypotheses pertaining to the movement ecology of Hawaiian gallinules (Table 1). These hypotheses were derived from expert opinion and literature on this and related taxa. We named these surfaces according to the datasets from which they were derived; the named groups are Elevation, Topographic Wetness Index (TWI), Land Use (LU), Roads, and Proximity‐to‐Water. To avoid issues with scaling across predictor variables, all surfaces were resampled to 30 m resolution and resistance was scaled from 1 to 100, where 1 is minimal resistance and 100 is maximum possible resistance. We assigned values to different landscape features based on expert opinion and available field evidence, with the objective of defining relative degrees of resistance (e.g., roads have higher resistance than agricultural fields), rather than specific numerical values (e.g., roads have a resistance value of 70, rather than 40) (Figure 3a–d) (see Spear et al., 2010; Zeller et al., 2012).

**Table 1:** Hypotheses of landscape effects on movement in Hawaiian gallinules and associated resistance surfaces, sources, and datasets for surface creation

Hypothesis	Resistance surface	Citation	Dataset
(1) Movement through low elevation	(1) *Elevation Two‐Class A*: Areas below 100 m have low resistance, higher elevations have high resistance; open ocean has high resistance	Perkins, 1903; Banko, 1987; M. Silbernagle (USFWS, ret.), pers. comm.	O`ahu Digital Elevation Model (DEM)
	(2) *Elevation Two‐Class B*: Areas below 100 m have low resistance, higher elevations have high resistance; open ocean has low resistance		
(2) Movement through low elevation, no sharp threshold	(3) *Elevation Linear A*: Landscape resistance increases linearly with elevation; open ocean has high resistance	Same as above	Same as above
	(4) *Elevation Linear B*: Landscape resistance increases linearly with elevation; open ocean has low resistance		
(3) Avoidance or higher cost to traversing steep terrain	(5) *Elevation Slope A*: Landscape resistance increases linearly with steepness of slope; open ocean has high resistance	M. Silbernagle (USFWS, ret.), pers. comm.	Same as above
	(6) *Elevation Slope B*: Landscape resistance increases linearly with steepness of slope; open ocean has low resistance		
(4) Movement through wet or mesic habitat, with a sharp threshold	(7) Topographic Wetness Index (*TWI*) [Link] *Two‐Class A*: Areas below threshold wetness value (11.5) have high resistance, areas above that have low resistance; open ocean has high resistance	van Rees & Reed, 2014; van Rees and Reed, unpubl. data	Same as above
	(8) *TWI Two‐Class B*: Areas below threshold wetness value have high resistance, areas above that have low resistance; open ocean has low resistance		
(5) Movement through wetter areas but no sharp threshold	(9) *TWI Linear A*: Landscape resistance decreases linearly with wetness; open ocean has high resistance	Same as above	Same as above
	(10) *TWI Linear B*: Landscape resistance decreases linearly with wetness; open ocean has low resistance		
(6) Avoidance or high cost to traversing urban areas	(11) Land Use (*LU*) [Link] *Two‐Class*: Urban land use areas have high resistance and all other land use types have low resistance	M. Silbernagle (USFWS, ret.), pers. comm.; Major, Johnson, King, Cooke, & Sladek, 2014;	NOAA LULC Dataset
	(12) *LU Three‐Class*: Urban land use areas have high resistance, wetland areas have low resistance, and all other land use types have moderate resistance		
(7) Movement through open areas, avoid closed areas	(13) *LU Structural*: Structurally open areas (agricultural fields, grassland, and wetland) have low resistance, intermediate areas (shrubland) have moderate resistance, covered areas (urban, forest) have high resistance	Keyel et al., 2012;	Same as above
(8) Graded ease of use	(14) *LU Full*: Wetlands have low resistance; other land types have increasing resistance in the following order: open land (grassland and agriculture), shrubland, forest, and urban	M. Silbernagle (USFWS, ret.), pers. comm.; Keyel et al., 2012; Major et al., 2014	Same as above
(9) Roads as barriers	(15) *Roads*: Large roads (highways) have maximum resistance, other roads have high resistance; all other areas have low resistance	K. Doyle (Hawaii DOFAW) pers. comm.	O`ahu Street Centerlines
(10) Proximity‐to‐Water (movement through riparian, drainage, and wetland corridors)	(16) *Water Binary*: Areas within a threshold distance value of water features have low resistance; all other areas have high resistance	Nagata, 1983; Takano & Haig, 2004	National Wetlands Inventory
	(17) *Water Linear 30‐m corridor*: Landscape resistance increases linearly with distance from water features and reaches maximum at 30 m		
	(18) *Water Linear 100‐m corridor*: Landscape resistance increases linearly with distance from water features and reaches maximum at 100 m		
	(19) *Water Linear 200‐m corridor*: Landscape resistance increases linearly with distance from water features and reaches maximum at 200 m		
	(20) *Water Negative Binomial*: Landscape resistance increases nonlinearly with distance from water features, but levels off		

^a^Topographic Wetness Index. ^b^Land use.

Elevation datasets were derived from 30 m resolution digital elevation models from the Hawaii Department of Commerce et al. (2007). Three types of surfaces were created using digital elevation models, with two versions each (based on the values assigned to open water, see below), for a total of six resistance surfaces (Table 1). The first of the elevation‐based hypotheses is binary models, in which we assigned a low resistance to all pixels below an empirically derived elevation threshold (resistance value = 10), and assigned a high value (80) to all pixels above that value (Hypothesis 1). The threshold (100 m) was based on the observation that most recorded occurrences and habitats of Hawaiian gallinules on O`ahu were at elevations below 100 m (U.S. Fish and Wildlife Service (USFWS), 1977; van Rees and Reed, unpubl. data). Birds could move through high elevations, but would move more readily (by a factor of 8) through low elevations. For linear resistance surfaces, we assumed a direct linear relationship between elevation and landscape resistance, with minimal resistance (resistance value = 1) at coastal elevations and maximum resistance (resistance value = 100) at maximum elevation for the island (Hypothesis 2). Finally, for slope surfaces, we assumed a linear relationship between degree of slope and resistance, where the maximum slope was given a value of 100, and flat ground was given a resistance value of 1 (Hypothesis 3).

Two additional hypotheses, with a total of four resistance surfaces, were based on the TWI (Beven & Kirkby, 1979). The TWI is a simple hydrological model that uses a digital elevation model (a spatial representation of elevation across a landscape) to approximate the likelihood that water would accumulate at any single point under uniform rainfall conditions. The TWI is a unitless measure, with higher values in areas that are likely to support standing water or mesic conditions, which are strongly associated with the occurrence of common gallinules and gallinule habitats (Bannor & Kiviat, 2002). We calculated TWI using the Geomorphology and Topology Toolbox (Evans & Oakleaf, 2011) in ArcGIS 10.2. In binary TWI models, we divided the landscape between low‐resistance pixels (resistance value = 1) at or above a threshold TWI value, and high‐resistance pixels (resistance value = 100) below that value (Hypothesis 4). We used a threshold value (TWI value = 11.5) for binary TWI surfaces based on van Rees and Reed (2014), who used TWI to infer the likely locations of historical wetlands on the Hawaiian Islands. This threshold TWI value was shown to divide extant wetlands from nonwetland areas. Accordingly, we took high TWI values to infer the presence of wet conditions and a high likelihood of wetland cover. Linear TWI models assumed an inverse linear relationship between TWI and landscape resistance, scaled from 0 to 100, where higher TWI values had lower resistance, and vice versa (Hypothesis 5).

Although most straight‐line pathways (the intervening space between two wetland habitats) between wetlands covered only terrestrial habitats, some pathways included portions of open ocean (see Figure 2), which might conceivably be crossed as gallinules moved between those habitats. The resistance of open ocean to Hawaiian gallinules is unknown, yielding no expert information on what resistance value to assign to waters along coastal regions in our resistance maps. Rallidae species have colonized oceanic islands all over the world, but are reluctant fliers when not dispersing (Ripley et al., 1977). Although genetic (Miller, Mullins, Haig, Takano, & Garcia, 2015) and observational (Takano & Haig, 2004; Worthington, 1998) evidence of long‐distance (~120 km) movements over open ocean water exist for the Marianas common moorhen, no movements of Hawaiian gallinules have been observed between the two islands they inhabit (Kaua`i and O`ahu, ~138 km apart), despite extensive mark–recapture efforts (Dibben‐Young, 2010; van Rees et al., 2018). There is accordingly some uncertainty with regard to the willingness of Hawaiian gallinules to disperse over open ocean. Consequently, we developed two scenarios each for the Elevation and Topographic Wetland Index hypotheses; version A, in which we assign high resistance (100) to open ocean water, and version B, where we assign lower resistance (20) to open ocean water.

Land use surfaces were created using the 2011 C‐CAP land cover dataset for O`ahu, (NOAA Ocean Service 2014), and these were used to model three hypotheses with regard to potential landscape effects on gallinule movement. In the binary land use model, we assigned low resistance (resistance value = 1) to all land cover types except for urban cover, which was assigned high resistance (resistance value = 100; Hypothesis 6). This binary urban resistance surface specifically pertains to the hypothesis that urban areas act as dispersal barriers to Hawaiian gallinules. In the land use three‐class surface, we assigned the same high value to urban areas, and low resistance value to wetland land cover, with a moderate resistance (resistance value = 50) to all other pixels. The three‐class land use surface thus integrates the hypotheses that wetland habitat may facilitate dispersal in Hawaiian gallinules with that of urban cover impeding dispersal. The structural land use model groups land use types into three categories along a gradient of habitat openness (cf. Keyel, Bauer, Lattin, Michael Romero, & Michael Reed, 2012), open (grassland, wetland, agricultural land, resistance value = 1), intermediate (shrubland, resistance value = 50), and closed (forest and urban, resistance value = 100). This structural land use surface reflects the hypothesis that gallinule movement may be affected by the physical structure of the landscape, either by physical or psychological mechanisms (e.g., Harris & Reed, 2002; Tremblay & St Clair, 2011; Trizio et al., 2005; Zeller et al., 2012) (Hypothesis 7). Finally, the full land use model combines all previous land use models and assigns different landscape resistance values for five habitat types: wetland, open areas, shrubland, forest, and urban, in order of increasing landscape resistance (resistance values = 1, 10, 30, 80, 100, respectively; Hypothesis 8).

The roads’ resistance surface was derived from the O`ahu Street Centerlines dataset (HOLIS, 2005) and assigns high landscape resistance values to highways and major roads, moderate values to all other roads, and low resistance to all other pixels. Our road‐based resistance surface addresses the hypothesis that roads impede dispersal of Hawaiian gallinules through either direct mortality or psychological inhibition (e.g., Benítez‐López, Alkemade, & Verweij, 2010; Thinh, Doherty, Bui, & Huyvaert, 2012; Zeller et al., 2012) (Hypothesis 9).

Finally, we derived resistance surfaces based on Proximity‐to‐Water from the National Wetlands Inventory dataset for Hawaii (USFWS, 2010) downloaded using the U.S. Fish and Wildlife Service's wetland mapper tool (http://www.fws.gov/wetlands/Data/Mapper.html). We excluded all large lacustrine (open water) wetlands, ocean shoreline wetlands, and estuarine marshes from this dataset because they are not used by Hawaiian gallinules (Banko, 1987), and we retained rivers, streams, freshwater low‐elevation wetlands, and other water features (drainage ditches and irrigation infrastructure). We then used the Euclidean distance tool to generate a raster dataset where each pixel was assigned a value based on its proximity to the nearest water feature. This category of resistance surfaces is based on anecdotal accounts that Hawaiian gallinules tend to travel along river margins, observations by the authors that the birds appear behaviorally inhibited from moving far from water, and evidence from related taxa that movement occurs along riparian corridors (Nagata, 1983; Takano & Haig, 2004; Hypothesis 10). In the binary Proximity‐to‐Water surface, we assigned a low resistance value (resistance value = 1) to all pixels within a distance of 30 m of a water feature and a high value (resistance value = 100) to all pixels outside of that radius. The threshold distance of 30 m was derived from observations by the authors of the distances at which Hawaiian gallinules are rarely seen away from water features. For the three Water Linear surfaces, we assumed a linear increase in landscape resistance with distance from a water feature that reaches its maximum at 30, 100, or 200 m from the water feature, respectively. Finally, for the Water Negative Binomial surface, we assumed a nonlinear relationship according to the negative binomial equation (Y = 100 – 4e^–(x–8)^), based on a function used by Trainor, Walters, Morris, Sexton, and Moody (2013) to describe potential effects of distance from habitat features affecting dispersal in another habitat specialist bird. The equation was parameterized using expert opinion to identify the minimum distances from a landscape feature at which landscape resistance would increase and at which increasing distance would cease to affect landscape resistance.

### 2.4 Effective and euclidean distances

Because little is known about the movement behavior of Hawaiian gallinules, we calculated effective distances among all pairwise combinations of occupied (and sampled) habitat patches using both cumulative least‐cost paths (Coulon et al., 2004; Michels et al., 2001) and resistance distances (McRae, 2006), which differ in their assumptions of an organism's movement behavior and knowledge of the surrounding landscape (Coulon et al., 2004). Circuit‐theory approaches simultaneously consider all potential movement pathways when calculating effective distances, while least‐cost path simulations consider only a single optimal pathway, thereby assuming that the animal has complete knowledge of the landscape. All distances were calculated among approximate centroids of each habitat patch, rather than between closest patch edges, because wetlands on O`ahu are small, isolated patches surrounded by large amounts of nonwetland matrix (van Rees & Reed, 2014). Consequently, the amount of within‐patch distance from centroid to patch edge is a negligible amount of total distance among centroids. We calculated least‐cost path distances using the cost‐distance function from the package gdistance (van Etten, 2017) in R (R Core Team, 2015) and resistance distances using Circuitscape 4.0 (McRae, Shah, & Edelman, 2016). We also calculated topographically adjusted Euclidean distances between population pairs using the near‐to‐table tool in ArcGIS.

### 2.5 Landscape genetic analyses

There is disagreement in the recent literature on which statistical methods are most appropriate for assessing the relationships between landscape features and genetic differentiation (Shirk, Landguth, & Cushman, 2017; Zeller et al., 2016). Consequently, following Balkenhol, Waits, and Dezzani (2009), we used three methods of analysis to reduce the potential for method‐dependent biases in our results. Datasets were analyzed in the form of distance matrices, with pairwise genetic distance (F_ST_) of microsatellite and mtDNA markers as the response variable and pairwise effective distance for a given model as the predictor variable. F_ST_ values were taken from van Rees, Reed et al. (2018), changing nonsignificant and negative values to zero. We used F_ST_ values from both microsatellites and mtDNA in all analyses. As in Phillipsen et al. (2015), we chose to compare only univariate models for our analysis, due to the high likelihood of collinearity between some of our resistance surfaces (e.g., TWI‐based vs. Proximity‐to‐Water).

We used simple Mantel tests (Legendre & Fortin, 2010; Mantel, 1967) to analyze landscape genetic relationships while accounting for the nonindependence of data point. Although Mantel tests have been criticized for having high type I error rates (Balkenhol et al., 2009; Graves, Beier, & Royle, 2013; Guillot & Rousset, 2013), they have been and continue to be widely used in the field of landscape genetics, so we included Mantel tests to make our results readily comparable to other research in the field. In addition to simple Mantel statistics, we also followed the causal modeling framework using partial Mantel tests (Cushman & Landguth, 2010; Cushman, McKelvey, Hayden, & Schwartz, 2006) and evaluated mean relative support ($\bar{\mathrm{RS}}$) for all models. Zeller et al. (2016) found that this approach performed well in selecting the best resistance surface in simulated landscape genetic studies, especially in highly fragmented landscapes like in this study. We performed all Mantel tests using the package vegan (Oksanen et al., 2016) in R.

We also analyzed landscape genetic relationships using the mixed‐model maximum‐likelihood population‐effects framework (MLPE; Clarke, Rothery, & Raybould, 2002; Van Strien, Keller, & Holderegger, 2012), following the methods described in Van Strien et al. (2012). This method accounts for the pairwise dependency of genetic and effective distance data by incorporating it into the covariance structure of the linear model, and accounting for it using a random effect, allowing differentiation from the fixed effects associated with predictor variables. Prior to model fitting, we scaled all predictor variables (effective distances) by dividing them by their maximum value, to avoid problems of scale mismatch during model fit, and to make model coefficients comparable and easy to interpret. We compared all resistance surface models using both Akaike's information criterion corrected for small sample size (AIC_C;_ Akaike, 2014; Hurvich & Tsai, 1989) and by calculating the $R_{\beta }^{2}$ statistic (Edwards, Muller, Wolfinger, Qaqish, & Schabenberger, 2008), which measures the proportion of observed variation explained by the fixed effects of the model, based on van Strien et al. (2014). We used both of these methods because they have each been criticized and recommended by different authors (Orelien & Edwards, 2008; Row, Knick, Oyler‐McCance, Lougheed, & Fedy, 2017; Van Strien et al., 2012; Verbeke, 1997), and we desired to use the advantages of each to make up for their respective shortcomings. Notably, recent work (Row et al., 2017) suggests that AIC performs well in model selection in simulated landscape genetic studies, avoiding biases that other methods may exhibit toward highly complex models. We fitted mixed effects models with REML estimation using the lmer function in the package lme4 (Bates, Maechler, & Bolker, 2011) in R, and calculated AIC values and generated AIC tables using AICtab function in the package AICcmodavg (Mazerolle, 2017). We calculated $R_{\beta }^{2}$ using the package pbkrtest (Halekoh & Højsgaard, 2014).

We implemented a post hoc analysis to test three additional models (see Supporting information Table S2) designed to account for three potential confounding factors that might have biased model selection toward Proximity‐to‐Water models. These were (a) that any resistance surface consisting of low‐resistance, linear features (corridors) outperforms all others, (b) that surrounding wetland habitat at source and destination nodes was driving patterns of simulated effective distance, and (c) that the spatial arrangement of features in the Proximity‐to‐Water resistance surfaces, and not their resistance values, was driving their ability to describe observed genetic structure. Scenario 1 was a special concern, considering that the Proximity‐to‐Water resistance surfaces were the only ones that contained linear features that could act as corridors. To test Scenario 1, we repeated our methods using an inverse version of the roads map, in which O`ahu's roads had low resistance, acting as corridors. For Scenario 2, we created a resistance surface identical to the Proximity‐to‐Water 100‐m buffer layer, but using a new dataset that only featured streams and drainage infrastructure, and from which all wetland areas had been removed. Finally, for Scenario 3, we tested the explanatory value of an inverse version of the Proximity‐to‐Water 100 m buffer resistance surface. These extra resistance surfaces were tested against our microsatellite genetic dataset.

## 3 RESULTS

### 3.1 Landscape genetic analysis

Models of the Proximity‐to‐Water group generally explained a higher amount of observed variation in pairwise population differentiation than any other group of models (Table 2). These models had consistently lower *p*‐values and higher r values in simple Mantel tests across both methods of estimating effective distance and had much higher $\bar{\mathrm{RS}}$ scores than all other models. The three highest Mantel's *r* values were 0.637, 0.562, and 0.530, belonging to the least‐cost‐path effective distances for Water Linear 100‐m corridor, Water Negative Binomial, and Water 30‐m corridor surfaces, respectively. Proximity‐to‐Water models also had the highest $\bar{\mathrm{RS}}$ values (0.650, 0.596, and 0.545 for least‐cost‐path simulations of Water Negative Binomial, Water Linear 30‐m corridor, and Water Linear 100‐m corridor, respectively). The three highest $R_{\beta }^{2}$ values were 0.343, 0.206, and 0.181, which corresponded to least‐cost‐path distance estimates of the Water Linear 100‐m corridor, Water Negative Binary, and Water Linear 30‐m corridor surfaces, respectively, and as with other metrics these highest values were from least‐cost‐path simulations of effective distance. The observed patterns of model support were consistent between genetic distances calculated using microsatellite and mitochondrial DNA (microsatellite results in Table 2, mitochondrial DNA in Supporting information). All observed statistically significant *p*‐values from simple Mantel tests were restricted to models from the Proximity‐to‐Water group. $R_{\beta }^{2}$ values of linear mixed models using the MLPE parameterization were also highest for Proximity‐to‐Water models, although the difference was less pronounced, and $R_{\beta }^{2}$ values were generally low. The Euclidean distance model performed poorly across all methods of comparison, and models from all groups except for Proximity‐to‐Water varied widely in performance across methods of estimating effective distance, but always performed more poorly than Proximity‐to‐Water models. We observed no clear pattern of model support between models with high resistance assigned to ocean water (A models; see Table 1) and models with low resistance assigned to ocean water (B models).

**Table 2:** Test statistics from Mantel (*r*) and partial Mantel tests, as well as mean relative support ($\bar{\mathrm{RS}}$) and $R_{\beta }^{2}$ values for all landscape resistance models evaluated using data on genetic differentiation (F_ST_ among 12 microsatellite loci) among 12 populations of Hawaiian gallinules on O`ahu

Model name (resistance surface)	Mantel r		Mantel *p*		$\bar{\mathrm{RS}}$		$R_{\beta }^{2}$	
	LCP	CS	LCP	CS	LCP	CS	LCP	CS
Elevation Two‐Class A	0.055	0.048	0.231	0.263	0.183	−0.075	0.082	0.075
Elevation Two‐Class B	0.014	0.075	0.386	0.255	−0.211	−0.054	0.065	0.085
Elevation Linear A	0.053	0.032	0.243	0.271	0.150	−0.243	0.082	0.064
Elevation Linear B	0.038	0.038	0.280	0.285	−0.240	−0.204	0.074	0.068
Elevation Slope A	0.054	0.128	0.233	0.096	0.132	0.129	0.081	0.083
Elevation Slope B	0.037	0.128	0.277	0.095	−0.251	0.139	0.073	0.083
TWI Two‐Class A	0.055	0.021	0.233	0.332	0.150	−0.353	0.083	0.053
TWI Two‐Class B	0.047	0.088	0.344	0.227	−0.254	−0.140	0.048	0.066
TWI Linear A	0.050	0.053	0.240	0.294	−0.094	−0.131	0.076	0.079
TWI Linear B	0.037	0.045	0.315	0.350	−0.205	−0.245	0.076,	0.077
LU Two‐Class	0.032	0.012	0.298	0.404	−0.402	−0.150	0.072	0.074
LU Three‐Class	0.040	0.190	0.284	0.126	−0.196	0.224	0.076	0.099
LU Structural	0.031	−0.066	0.302	0.596	−0.266	−0.443	0.075	0.056
LU Full	0.084	0.015	0.144	0.436	0.305	−0.158	0.102	0.074
Roads	0.036	0.100	0.275	0.198	−0.348	0.061	0.072	0.085
Water Binary	0.368	0.375	0.009*	0.046*	0.522	0.470	0.131	0.132
Water Linear 30‐m Corridor	0.530	0.281	0.011*	0.069	0.596	0.396	0.181	0.115
Water Linear 100‐m Corridor	0.637	0.273	0.024*	0.092	0.545	0.341	0.343	0.114
Water Linear 200‐m Corridor	0.313	0.219	0.009*	0.102	0.445	0.287	0.122	0.107
Water Negative Binomial	0.562	0.251	0.015*	0.074	0.650	0.355	0.206	0.111
Euclidean Distance^a^	0.026		0.317		−0.458		0.069	

Notes. For each model, statistics are given separately for effective distances calculated using cumulative least‐cost path (LCP) and resistance distances in Circuitscape (CS). The Euclidean distance model did not include effective distance, so only one value is presented for each statistic, with the exception of partial mantel $\bar{\mathrm{RS}}$, where mantel r values were compared to those from models run with effective distances calculated using both methods. Asterisks (*) indicate statistically significant *p*‐values at the α = 0.05 level. TWI and LU stand for Topographic Wetness Index and Landscape Use, respectively.

^a^Only one column per statistical method, because Euclidean distance cannot be simulated.

Among Proximity‐to‐Water models, *r* values, $\bar{\mathrm{RS}}$ values, and $R_{\beta }^{2}$ values were consistently higher using effective distances calculated with cumulative least‐cost paths than those calculated in Circuitscape. While all Proximity‐to‐Water models using least‐cost‐path had statistically significant *p*‐values, only one, the binary model, had a significant *p*‐value among those with effective distances measured in Circuitscape. The best overall models differed according to both measure of effective distance and method of statistical analysis, with the two‐class, linear to 100 m distance, linear to 200 m distance, and negative binomial distance functions scoring highest for at least one statistic and effective distance measure.

Our AIC_C_ analysis of MLPE‐parameterized linear mixed models showed clear support for Proximity‐to‐Water models, with nine of the top 10 models being based on Proximity‐to‐Water surfaces (Table 3). The least‐cost‐path simulated versions of models performed better in our AIC analysis as well, with the top five models coming from our least‐cost‐path datasets, and only three of the top ten models coming from effective distances measured in Circuitscape. The second‐ranked model had an ΔAIC_C_ > 4 from the top model, indicating a substantial difference in support from the top model (Burnham & Anderson, 2002). The β (coefficient or slope) estimates for the fixed effect of the top five univariate models were 0.51, 0.35, 0.30, 0.19, and 0.15.

**Table 3:** ΔAIC_C_ values and Akaike weights for the top 10 linear mixed‐models relating effective distance to genetic differentiation in Hawaiian gallinules on O`ahu

Model name (resistance surface & simulation method)	ΔAIC_C_	AIC_C_ weight
Water Linear 200‐m Corridor (LCP)	0.0	0.76
Water Negative Binomial (LCP)	4.16	0.10
Water Linear 100‐m Corridor (LCP)	5.60	0.05
Water Binary (LCP)	8.93	<0.01
LU Full (LCP)	9.15	<0.01
Water Binary (CS)	9.26	<0.01
Water Linear 300‐m Corridor (LCP)	9.48	<0.01
Water Linear 200‐m Corridor (CS)	10.52	<0.01
Water Linear 100‐m Corridor (LCP)	10.60	<0.01
Water Negative Binomial (CS)	10.96	<0.01

Note. Models were parameterized using the MLPE design from Clarke et al. (2002) to account for the lack of independence of pairwise data. The simulation mode by which effective distance was calculated for each model (least‐cost paths—LCP or Circuitscape—CS) is listed after the model name. TWI and LU stand for Topographic Wetness Index and Landscape Use, respectively.

Among our post hoc tests, the roads‐as‐corridors’ resistance surface performed very poorly overall, with a low $\bar{\mathrm{RS}}$ and $R_{\beta }^{2}$ value, although the mantel *p*‐value for effective distances created in Circuitscape was near significance (Mantel *r* = 0.182, *p* = 0.07). The poor performance of this resistance surface using our least‐cost‐path algorithm implied that linear features alone cannot explain the high performance of Proximity‐to‐Water models using least‐cost‐path effective distances. The streams and drainage surface with wetlands removed performed comparably to other Proximity‐to‐Water models, with a high Mantel *r* value (0.474 for LCP, 0.327 for CS), and lower Mantel *p* (0.021 for LCP, 0.058 for CS), and high $\bar{\mathrm{RS}}$. Although this model did perform more poorly than other Proximity‐to‐Water resistance surfaces, its sustained high performance compared to other resistance surfaces supports our initial interpretation that Proximity‐to‐Water surfaces are explaining gene flow on a landscape context, and not simply because they include local habitat features at population nodes. Finally, our inverse “water as barrier” resistance surface performed extremely poorly, indicating that the resistance values assigned to the original Proximity‐to‐Water surfaces are indeed responsible for high model performance.

## 4 DISCUSSION

To our knowledge, this is the first landscape genetic analysis for a terrestrial waterbird species. As such, it represents a step toward overcoming one bias in the growing literature of landscape genetics (Kozakiewicz et al., 2017; Zeller et al., 2012). We found consistent support for resistance surfaces that were based on Proximity‐to‐Water, while all other resistance surfaces showed low explanatory value and statistical significance. The higher explanatory power and statistical significance of Proximity‐to‐Water surfaces was robust across four model selection metrics (Mantels r and *p*‐value, $\bar{\mathrm{RS}}$, $R_{\beta }^{2}$, and AIC_C_), two simulation frameworks (least‐cost paths and resistance distance), and two genetic marker types, suggesting that the presence of water features explains 10.7%–63.7% of variation in observed genetic structure among Hawaiian gallinule populations inhabiting wetlands on O`ahu (Table 2). Although the results of simple Mantel tests should be interpreted cautiously (Balkenhol et al., 2009; Zeller et al., 2016), we see congruent patterns in several more robust metrics. Zeller et al. (2016) found that simple Mantel's r and $\bar{\mathrm{RS}}$ performed best when comparing resistance surfaces where landscapes were highly fragmented, and we believe our study system fits this condition well. We also analyzed our results using linear mixed effects models fit with MLPE, which is currently considered the best performing method for performing regressions on matrix data (Shirk et al., 2017), and had similar results using two different methods of model selection. The consistency across model selection metrics and sharp contrast in support compared to all other models of landscape resistance provide evidence that the presence of small wetlands, drainage canals, and streams enhances genetic connectivity in this endangered subspecies. Our findings support suggestions by other authors that Hawaiian gallinules may move along river systems or other linear water features (Nagata, 1983; van Rees & Reed, 2015). The potential use of linear water features as dispersal corridors by Hawaiian gallinules coincides with observations in other tropical birds (e.g., Gillies & St. Clair, 2008; Sekercioglu, 2009; Takano & Haig, 2004). The results of our post hoc tests lend additional credence to our findings, but a biological, mechanistic explanation for the phenomenon is also important to consider.

One potential mechanism may be a “landscape of fear” (Laundré, Hernández, & Ripple, 2010); in this case where Hawaiian gallinules perceive lower predation risk near water features and accordingly are more willing to travel along them. Reduced antipredator behavior near water features has been documented in other rail taxa (Dear, Guay, Robinson, & Weston, 2015), and rails and other waterbirds tend to flee toward water as part of their normal predator escape behavior (Lima, 1993). Perceiving lower risk due to ease of escape, Hawaiian gallinules may accordingly experience fewer behavioral barriers to movement when closer to water features (sensu Harris & Reed, 2002).

**Figure 4:**
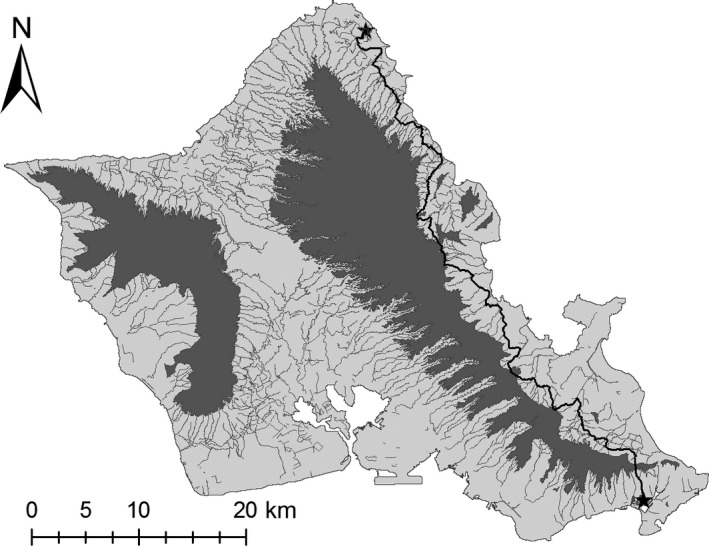
Approximation of least‐cost pathway between James Campbell National Wildlife Refuge and Keawawa Wetland, calculated using the 100‐m corridor distance‐to‐water resistance surface and the least‐cost path tool in ArcGIS

**Figure 5:**
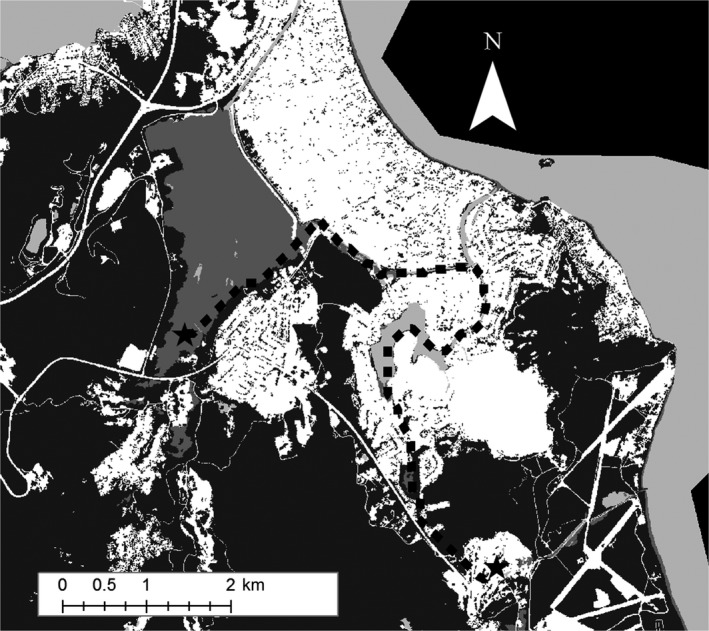
Approximation of least‐cost pathway between Kawainui Marsh and Olomana Golf Links, calculated using the 100‐m corridor distance‐to‐water resistance surface and the least‐cost path tool in ArcGIS. For illustrative purposes, the path has been projected over a modified version of the NOAA C‐CAP 2011 map of O`ahu, showing urban areas in white and undeveloped areas in dark gray, with water features in medium gray and open water in light gray

The predictions of our Proximity‐to‐Water models make intuitive sense based on expert opinion and limited observations of Hawaiian gallinule dispersal behavior. Visual inspection of least‐cost paths developed using our 100‐m corridors, Proximity‐to‐Water surface, and the least‐cost‐path function in ArcGIS (Figures 4 and 5) shows that predicted movement paths following water features result in avoidance of mountains and high elevation features at a large scale, and urban features at a small scale, despite not explicitly including those features in resistance surfaces. For example, a pathway between James Campbell National Wildlife Refuge and Keawawa wetland (Figure 4) involves traveling along the coastline and parallel to the Ko`olau mountains, rather than over the mountains, and the least‐cost‐path between Kawainui marsh and the Olomana golf links makes use of extensive drainage infrastructure and nearby streams, avoiding direct passage through urban areas (Figure 5). Thus, the Proximity‐to‐Water layers implicitly feature other aspects of gallinule movement ecology observed anecdotally, specifically no observations at high elevation and a high susceptibility to road mortality in urban environments.

Because model rankings are mixed between different Proximity‐to‐Water models across different criteria of model selection, we cautiously refrain from selecting one of those models as being the best supported overall. Given the limited genetic variation exhibited by the subspecies (van Rees, Reed et al., 2018), and impacts of a recent population bottleneck on genetic diversity within Hawaiian gallinules (Sonsthagen, Wilson, & Underwood, 2017), it could be that our current sample is insufficient to distinguish between functions relating Proximity‐to‐Water to resistance values. Additional factors affecting dispersal in other taxa (e.g., conspecific attraction; Smith & Peacock, 1990; Serrano & Tella, 2003) may also influence dispersal rates in Hawaiian gallinules, but were not explored in this study. While genetic structure was detected at small spatial scales, addition of whole genomic or reduced representation genomic data may provide greater spatial resolution and increase our ability to detect landscape features that are influencing gallinule movement patterns (Kozakiewicz et al., 2017; Szulkin, Gagnaire, Bierne, & Charmantier, 2016). Finally, Zeller et al. (2016) note that model selection criteria for resistance surfaces perform very well at selecting the best among competing models, but not necessarily in estimating precise parameter values. Consequently, we do not attempt to quantify the relationship between Proximity‐to‐Water and landscape resistance, but value the acquired results as among the first empirical evidence describing the movement behavior of gallinules (but see Takano & Haig, 2004).

Consistently higher correlation coefficients and greater statistical significance among models that were applied using least‐cost‐path effective distances implies that the random walk model (upon which Circuitscape resistance distances are based McRae et al., 2014) performs poorly in simulating movements of Hawaiian gallinules on O`ahu, and that birds may navigate the island with some degree of knowledge of their larger landscape context. The use of prior landscape knowledge may be typical of waterbirds, which often conduct long‐distance dispersal by means of higher‐altitude flights, thereby providing a view of a larger portion of the landscape. Furthermore, the finding that models based on road mortality, slope, elevation, and difficulty of traversing urban areas performed poorly may provide additional support for the hypothesis that Hawaiian gallinules are not dispersing on foot, despite being highly cursorial, and rarely seen in flight. The hypothesis of flying dispersal in gallinules is supported by observations from Taylor and Anderson (1973), who reported 11 common gallinules (*G. g. cachinnans*) that were killed after striking a television transmission tower (~430 m above ground level) in central Florida during nocturnal flights. It is worth noting that the tower was near “a small lake drained by a 20‐foot wide canal; both contained water during the kills.” Also of interest is that kills occurred at night, supporting other observations that common gallinules and common moorhen (*G. chloropus*) perform higher‐altitude flights at night (Roselaar, 1980; Taylor, 2010), which may explain why long flights are rarely observed on O`ahu.

Although our analysis yielded a strong and consistent signal that water features decrease landscape resistance to movement for Hawaiian gallinules on O`ahu, several important limitations to our study are worth noting. First, although genetic data are a useful descriptor of overall gene flow as a result of dispersal, they ultimately represent only a portion of animal movements (Spear et al., 2010), specifically movements that lead to interwetland dispersal and successful breeding (Cushman, Lewis, & Landguth, 2014). Accordingly, although they match limited observations in related taxa (Takano & Haig, 2004), and anecdotal observations in this subspecies (Nagata, 1983), results from this study do not necessarily reflect the actual behavioral decisions made by individual Hawaiian gallinules as they traverse the island landscape. Additionally, the reliance of this study on the testing of expert‐designed models against empirical data and the subsequent process of model selection limits results to the best model among those chosen, and not necessarily the best possible model for describing gene flow in Hawaiian gallinules (cf. Beissinger & Snyder, 2002).

### 4.1 Conservation Implications and Priorities for Future Work

Empirical studies on the movement behavior of Hawaiian gallinules will be important to validate the results of our landscape genetic analysis and investigate the fine‐scale behavioral decisions that lead to the observed population‐level patterns of gene flow. More consistent mark–resighting efforts (Dibben‐Young, 2010) and study methods with higher spatiotemporal resolution will be important steps for improving our knowledge of movement behavior in this subspecies. GPS dataloggers and transmitters have been used to great effect in tracking a number of bird species (Gagliardo, Ioalè, Savini, Lipp, & Dell'Omo, 2007; Rodríguez et al., 2012), and translocation studies like that of Gillies and St. Clair (2008) allow for experimental manipulation of dispersal direction and matrix type.

An important implication of this study is that habitats formerly hypothesized to have little value to Hawaiian gallinules (e.g., drainage ditches and canals, forested and vegetated streams, and roadside swales) may actually affect their population persistence by increasing population connectivity. The management dependency of Hawaiian gallinules (Reed et al., 2012ab,) has led to the assumption that unmanaged wetlands and riparian systems are of little to no value for them. This study suggests that such unmanaged water features act as corridors, which may increase population persistence in fragmented landscapes by alleviating problematic consequences of isolation (Gilbert‐Norton, Wilson, Stevens, & Beard, 2010). As van Rees and Reed (2015) speculated, shifts in water management toward a greater emphasis on green stormwater infrastructure might simultaneously provide conservation benefits by creating such corridors for waterbirds like the Hawaiian gallinule. Such landscape changes would represent a gain for both the management of imperiled water resources (Giambelluca, 1986; Ridgley & Giambelluca, 1991) and threatened wildlife on O`ahu.

## CONFLICT OF INTEREST

None declared

## AUTHOR CONTRIBUTION

Charles van Rees conducted statistical and spatial analyses and simulations and wrote the manuscript. J. Michael Reed provided advice on statistical analyses, contributed to the writing of the manuscript, and supervised the organization of the project. Robert E. Wilson reviewed and contributed to the writing of the manuscript and provided guidance throughout collection and analysis of genetic data. Jared G. Underwood supervised sample collection and field logistics, and contributed to the writing of the manuscript. Sarah A Sonsthagen supervised collection and analysis of genetic data, provided guidance on interpretation of genetic results, and contributed to the writing of the manuscript.

## DATA ACCESSIBILITY

Data from this study (matrices of genetic distances, resistance surfaces in analysis, and effective distance matrices) available from the Dryad Digital Repository: https://doi.org/10.5061/dryad.p90b87p.
