# Supplementary material for: Landscape genetics identifies streams and drainage infrastructure as dispersal corridors for an endangered wetland bird
Source: Ecol Evol. 2018 Jul 24;8(16):8328–43. doi: 10.1002/ece3.4296 (PMC6145004; doi:10.1002/ece3.4296)
Supplement: Supplementary file 1 [file ECE3-8-8328-s001.docx]

| Table S1: Test statistics from mantel and partial mantel tests, as well as $R_{\beta}^{2}$ values for all landscape resistance models evaluated using data on genetic differentiation (F_ST_ among 520bp sequences of ND2 region of mtDNA) among 12 populations of Hawaiian gallinules on Oahu. For each model, statistics are given separately for effective distances calculated using Cumulative Least-cost Path (LCP) and resistance distances in Circuitscape (CS). The Euclidean distance model did not include effective distance, so only one value is presented for each statistic, with the exception of partial mantel $\bar{RS}$, where mantel r values were compared to those from models run with effective distances calculated using both methods. Asterisks (*) indicate statistically significant p-values at the α = 0.05 level. | | | | | | | | |
| --- | --- | --- | --- | --- | --- | --- | --- | --- |
| Model Name | Mantel r | | Mantel p | | $\bar{RS}$ | | $R_{\beta}^{2}$ | |
|  | LCP | CS | LCP | CS | LCP | CS | LCP | CS |
| Elevation Binary A | 0.033 | 0.026 | 0.279 | 0.309 | 0.157 | -0.160 | 0.077 | 0.0698 |
| Elevation Binary B | -0.029 | 0.030 | 0.540 | 0.34 | -0.219 | -0.126 | 0.059 | 0.076 |
| Elevation Linear A | 0.027 | 0.017 | 0.301 | 0.308 | 0.073 | -0.326 | 0.076 | 0.062 |
| Elevation Linear B | 0.010 | 0.012 | 0.362 | 0.350 | -0.278 | -0.286 | 0.068 | 0.064 |
| Elevation Slope A | 0.038 | 0.132 | 0.273 | 0.097 | 0.162 | 0.185 | 0.077 | 0.085 |
| Elevation Slope B | 0.021 | 0.132 | 0.3275 | 0.0959 | -0.181 | 0.159 | 0.069 | 0.085 |
| TWI Binary A | 0.038 | 0.032 | 0.268 | 0.291 | 0.178 | -0.278 | 0.078 | 0.057 |
| TWI Binary B | 0.012 | 0.095 | 0.444 | 0.213 | -0.117 | -0.052 | 0.052 | 0.069 |
| TWI Linear A | 0.027 | 0.046 | 0.287 | 0.315 | -0.058 | -0.184 | 0.073 | 0.072 |
| TWI Linear B | -0.017 | 0.031 | 0.500 | 0.389 | -0.222 | -0.289 | 0.067 | 0.067 |
| LU Binary | 0.018 | 0.036 | 0.343 | 0.379 | -0.338 | -0.161 | 0.066 | 0.067 |
| LU Three-Class | 0.022 | 0.21 | 0.332 | 0.097 | -0.173 | 0.302 | 0.069 | 0.100 |
| LU Structural | 0.020 | -0.051 | 0.335 | 0.582 | -0.197 | -0.273 | 0.070 | 0.066 |
| LU Full | 0.072 | 0.043 | 0.184 | 0.372 | 0.358 | -0.008 | 0.096 | 0.080 |
| Roads | 0.019 | 0.081 | 0.338 | 0.252 | -0.287 | 0.018 | 0.067 | 0.076 |
| Water Binary | 0.323 | 0.347 | 0.013* | 0.051 | 0.518 | 0.509 | 0.122 | 0.137 |
| Water Linear 30m Corridor | 0.454 | 0.265 | 0.016* | 0.072 | 0.555 | 0.426 | 0.158 | 0.116 |
| Water Linear 100m Corridor | 0.535 | 0.248 | 0.025* | 0.096 | 0.458 | 0.326 | 0.247 | 0.112 |
| Water Linear 200m Corridor | 0.277 | 0.216 | 0.013* | 0.101 | 0.459 | 0.337 | 0.115 | 0.108 |
| Water Negative Binomial | 0.476 | 0.240 | 0.018* | 0.080 | 0.565 | 0.383 | 0.170 | 0.111 |
| Euclidean Distance | 0.008 | | 0.378 | | -0.395 | -0.407 | 0.063 | |

| Table S2: Test statistics from mantel and partial mantel tests, as well as $R_{\beta}^{2}$ values for validation landscape resistance models evaluated using data on genetic differentiation (F_ST_ among 12 microsatellite loci) among 12 populations of Hawaiian gallinules on Oahu. For each model, statistics are given separately for effective distances calculated using Cumulative Least-cost Path (LCP) and resistance distances in Circuitscape (CS). The Euclidean distance model did not include effective distance, so only one value is presented for each statistic, with the exception of partial mantel $\bar{RS}$, where mantel r values were compared to those from models run with effective distances calculated using both methods. Asterisks (*) indicate statistically significant p-values at the α = 0.05 level. | | | | | | | | |
| --- | --- | --- | --- | --- | --- | --- | --- | --- |
| Model Name | Mantel r | | Mantel p | | $\bar{RS}$ | | $R_{\beta}^{2}$ | |
|  | LCP | CS | LCP | CS | LCP | CS | LCP | CS |
| Facilitation by Roads | -0.073 | 0.1816 | 0.641 | 0.07 | -0.7992 | -0.148 | 0.04 | 0.06 |
| Water as barrier | 0.074 | -0.3906 | 0.253 | 0.932 | -0.338 | -0.531 | 0.06 | <0.001 |
| Water 100m corridor – all wetlands removed | 0.474 | 0.327 | 0.003* | 0.058 | 0.507 | 0.260 | 0.14 | 0.09 |
| Distance to Water (Best of original models) | 0.552 | 0.430 | 0.004* | 0.021* | 0.6305 | 0.42 | 0.17 | 0.1108 |
